# Development of an embedded system-based automatic timing reminder device for improving autoclave cooling period management

**DOI:** 10.1371/journal.pone.0326899

**Published:** 2025-08-12

**Authors:** Yu Meng, Ping Gui, Shunlin Tan, Wei Zheng, Hao Wang

**Affiliations:** 1 Sterile Processing Department, Sichuan Tianfu New Area People’s Hospital, Chengdu, China; 2 Sterile Processing Department, Sichuan Clinical Research Center for Cancer, Sichuan Cancer Hospital &Institute, Sichuan Cancer Center, Affiliated Cancer Hospital of University of Electronic Science and Technology of China, Chengdu, China; PLOS: Public Library of Science, UNITED KINGDOM OF GREAT BRITAIN AND NORTHERN IRELAND

## Abstract

**Purpose:**

The study was conducted with the aim of developing an automatic timing reminder device based on embedded systems to manage the cooling period of sterilized items autoclave in the Sterile Processing Department (SPD). The primary objective was to reduce the wet packs incidence rate and potential contamination risks by ensuring that sterilized items are allowed to cool for a minimum of 30 minutes before handling.

**Materials & methods:**

The device, which utilizes the ATMEGA328 as the central control chip, incorporates a diffuse reflection type photoelectric sensor switch and encompasses modules for storage, power supply, speaker, display screen, real-time clock, and working indicator lights. It was programmed with Arduino 1.8.19 software to initiate timing upon sensor obstruction and emit reminders at predetermined intervals. Installed on a sterilizer cart using plastic cable ties, the device was tested over a 4-week period against a control group that relied on traditional methods for determining cooling time. The study monitored cooling time compliance rate, wet packs incidence rate, the frequency with which SPD technicians touched sterilized items, and technician satisfaction through a self-designed survey.

**Results:**

The experimental group, using the timing reminder device, achieved a significantly higher cooling time compliance rate of 92.93% compared to the control group’s 72.92%. The wet packs incidence rate in the experimental group was markedly lower at 0.18%, contrasted with the control group’s 0.49%. Furthermore, the number of times SPD technicians touched sterilized items in the experimental group was significantly reduced compared to the control group. Additionally, the satisfaction score in the experimental group was notably higher than that of the control group.

**Conclusion:**

The automatic timing reminder device, developed based on embedded systems technology, demonstrates good cost-effectiveness and high satisfaction among SPD technicians. It effectively standardizes the management process of autoclave. Therefore, the device holds significant potential for widespread application in other SPDs. Future research will further explore the potential applications of embedded systems in the healthcare field to expand its value in various medical procedures.

## Introduction

The Sterile Processing Department (SPD) is tasked with the cleaning, disinfection, and sterilization of reusable medical instruments and equipment for various wards, playing a pivotal role in the prevention and control of nosocomial infections [1]. Sterilization techniques can be categorized based on temperature into high-temperature and low-temperature methods. Dry heat sterilization and autoclave are among the high-temperature sterilization [2,3]. Ethylene oxide gas sterilization, hydrogen peroxide gas sterilization, and low-temperature steam formaldehyde are some of the more frequently employed low-temperature sterilization methods [4–6]. Autoclave technology is highly advanced, offering superior sterilization efficacy, cost-effectiveness, and the absence of chemical residue, making it the preferred method for heat-resistant medical devices [7,8]. Whether in developed nations such as the United States, the United Kingdom, and Germany, or in developing countries across Asia and South America, autoclaves are the cornerstone of SPD’s sterilization equipment. However, despite its effectiveness, challenges remain in ensuring proper cooling periods after sterilization to prevent issues such as wet packs.

A wet pack is defined as visible moisture on the interior or exterior of a package following sterilization and the appropriate cooling period. Wet packs pose a significant concern as the moisture can facilitate the migration of microorganisms from the exterior to the interior of the package, rendering the package a sterilization failure. If wet packs are identified in the processing area, they must not be released. Similarly, if discovered in the user area (e.g., operating room), they should not be used [8]. Consequently, wet packs can escalate the cost of reprocessing medical trays, disrupt the order of medical work, and lead to rust and contamination of instruments [9].Improving the quality of saturated steam, extending the drying time in the procedure, and reasonable loading of instrument trays can reduce the occurrence of wet packs [10,11]. In addition, waiting for sufficient cooling time after sterilization before unloading can also decrease the wet packs incidence rate. Industry guidelines from the World Health Organization [7], the United States [8], and Belgium [12] all recommend that sterilized items be cooled to room temperature before being moved and handled. According to Chinese health industry standards, the cooling period for sterilized items after the sterilization cycle should not be less than 30 minutes [13]. However, in the practical work of SPDs in China, due to the lack of appropriate tools to calculate the cooling time, it is common for sterilized items to be unloaded before the cooling time is sufficient. More seriously, some SPD technicians always use the back of their hands to touch sterilized items to judge whether they have cooled down to a temperature that allows for unloading. These practices increase the occurrence of wet packs and significantly increase the risk of contamination of sterilized items.

An infrared gun or temperature-sensing device may be used to verify that sterilized items have reached a defined temperature [8]. However, infrared guns exhibit significant limitations when measuring the surface temperature of items with varying packaging materials and volumes. First, differences in emissivity among packaging materials directly affect measurement accuracy. For example, metals (particularly those with smooth surfaces) exhibit low emissivity (0.1–0.3), leading to strong reflections of ambient radiation and resulting in measured values significantly lower than the true temperature [14,15]. Second, small or curved items suffer from incomplete coverage by the infrared spot and interference from background radiation, making it challenging to precisely target the measurement area. Additionally, when measuring sterilized items, infrared guns theoretically require manual adjustment of emissivity settings based on material properties. Failure to calibrate these settings may introduce errors exceeding several tens of degrees Celsius, severely compromising measurement accuracy [16,17].

These limitations highlight the need for a simple cooling period management technology that relies on time rather than temperature as the criterion for judgment. Manual timers can be used to measure cooling time without relying on the temperature of the item. However, using a manual timer requires the SPD technician to manually start and stop it multiple times, and its alarm sound cannot be customized. Furthermore, any recorded data cannot be stored. Some SPDs utilize medical device full-life-cycle management software, which can be configured to release sterilized items only after a specified period following the sterilization cycle. Scanning the label of a sterilized item before the cooling time reaches the set duration will trigger an error message. However, many SPDs currently using such software lack this functionality. Additionally, the action of unloading sterilized items onto metal shelves is sometimes not synchronized with the software system’s operations, which limits the practical application of this feature.

Embedded technology, which integrates hardware and software systems into host devices, enhances work efficiency and intelligence while reducing the need for manual operations. It also provides robust data storage capabilities, making it widely applicable across various fields [18,19]. Given the convenience and effectiveness of embedded technology, we have designed and fabricated a sterilized items cooling period automatic reminder device that operates without the need for conscious intervention. The primary innovation of this embedded technology-based device lies in its automation, which eliminates the need for sterilization personnel to perform separate operations for time recording, music playback, and data storage. This represents a significant improvement over manual timers.

## Materials and methods

### Device fabrication

**Fig 1:**
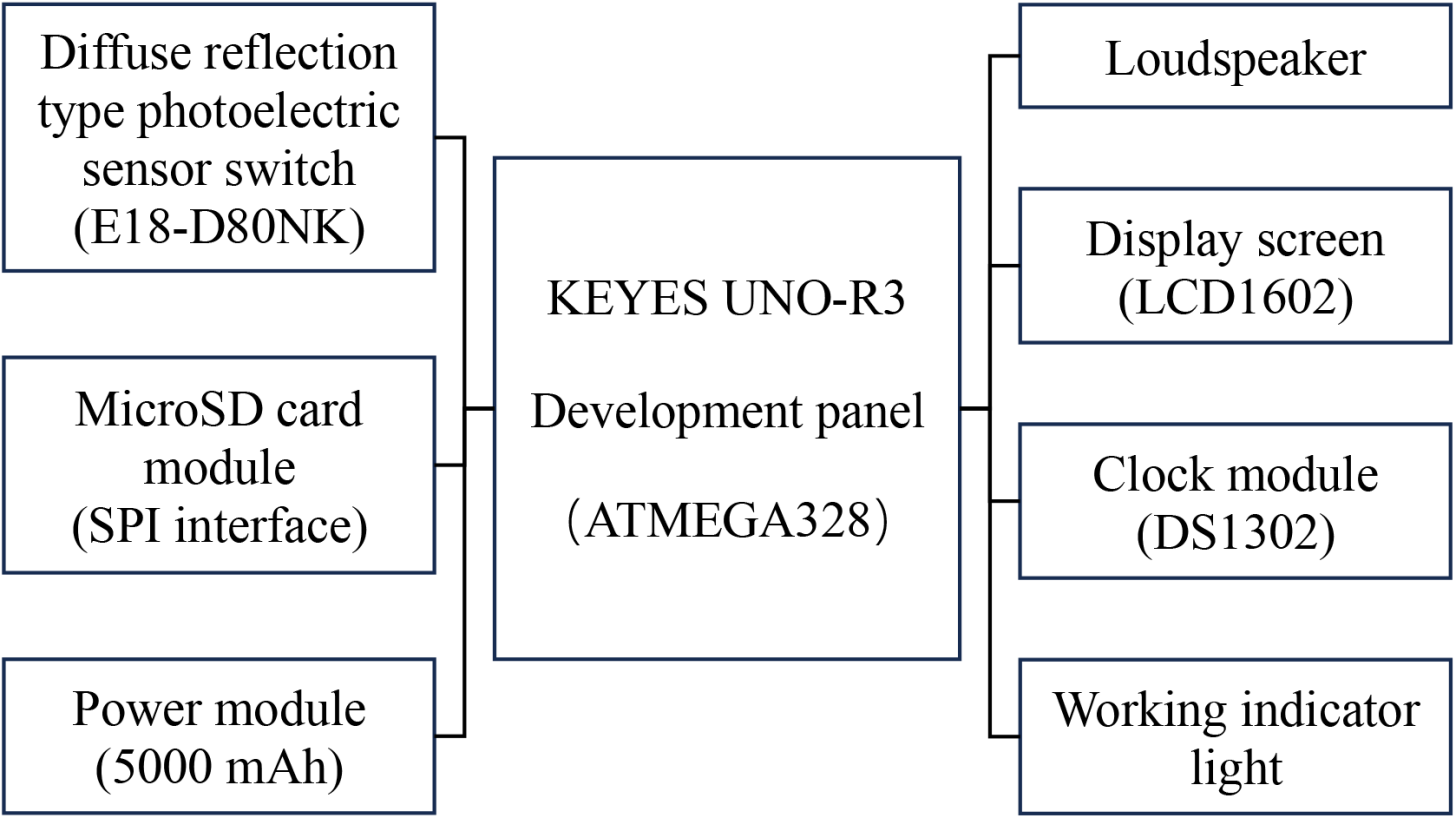
Structure framework diagram of the automatic timing reminder device. The diffuse reflection type photoelectric switch serves as a sensor to start and stop the device. The volume of the loudspeaker can be adjusted via a knob, and the display screen shows the date, current time, and cooling time.

**Fig 2:**
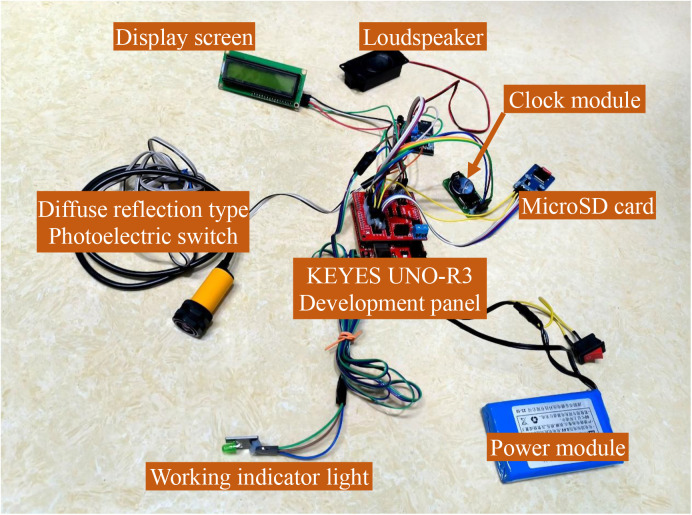
Photograph of the automatic timing reminder device. The power cables connecting the diffuse reflection type photoelectric switch and the working indicator light to the development board are each over 1 meter in length, enhancing the device’s compatibility with various sterilizer carts.

The automatic timing reminder device for improving autoclave cooling period management, employing the ATMEGA328 as the primary control chip, integrates a diffuse reflection type photoelectric sensor switch (specification is a three-wire NPN normally open type, with a sensing distance of 3 cm to 80 cm, switching to the “on” state when obstructed by an object and sending an “off” signal when unobstructed) as the signal acquisition module. The sensor operates by emitting and receiving infrared rays. The indoor lighting contains only a minimal amount of infrared, which is insufficient to trigger the sensor. Tiny dust particles can only reflect a negligible amount of infrared, thus they won’t activate the device’s timing function. Furthermore, the SPD work area has very little dust to begin with. Additionally, this sensor boasts the advantages of compact size, easy replacement, and low cost (only $2). The device is also equipped with modules for storage, power supply, speaker, display screen, real-time clock, and working indicator lights (Figs 1 and 2).

**Fig 3:**
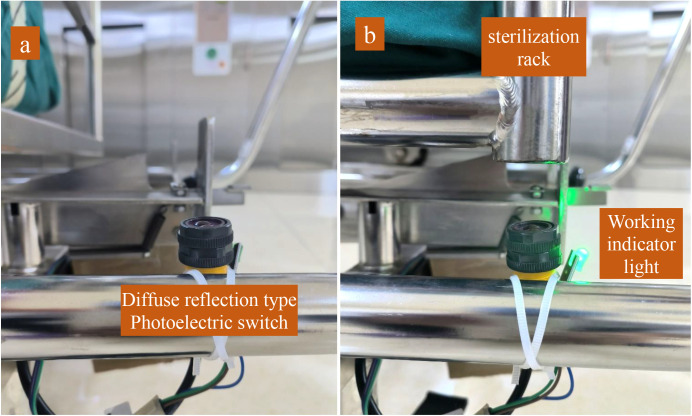
Working principle of the diffuse reflection type photoelectric switch. The diffuse reflection type photoelectric switch is a sensor that integrates both infrared emission and reception. When an obstruction is present above the sensor, the emitted infrared light is reflected, and the sensor detects the reflected light to activate the working state. a) Without a sterilization rack, the sensor remains inactive, and the device is in standby mode. b) With a sterilization rack, the sensor activates the device to start timing, and the working indicator light turns on and remains lit.

**Fig 4:**
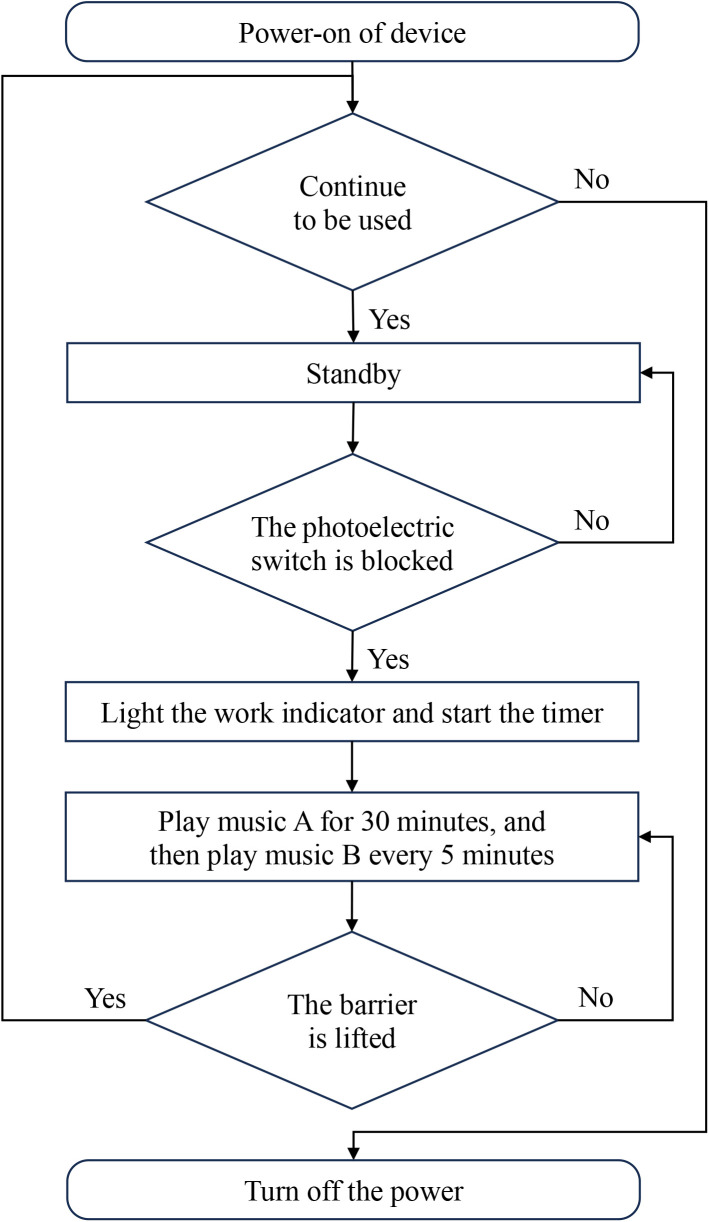
Software flow chart of the automatic timing reminder device.

The program for this timing reminder device is developed using Arduino 1.8.19 embedded software. Upon startup, the device’s display shows the time in the China Standard Time Zone (UTC + 8). When an object obstructs the path in front of the photoelectric switch, it sends a signal “on”, the working indicator light illuminates, and the display screen begins timing from 00:00. After reaching 30 minutes, the loudspeaker emits a reminder music A once (lasting 30 seconds), and the display screen continues to count time, emitting a reminder music B every 5 minutes thereafter (lasting 3 seconds). We consider a 5-minute interval to be more reasonable, as 1-minute and 2-minute intervals are too short, while 10 or 15-minute intervals would be too long. The device continues to count time and provide intermittent reminders until the obstructing object is removed, at which point the photoelectric switch sends a signal “off”, concluding the cycle. The working indicator light turns off, timing ceases, and data is stored. The display enters standby mode, awaiting the initiation of the next cycle (Figs 3 and 4). During the cooling period following autoclave, the rate at which temperature declines varies among different medical instrument trays, textiles, and rigid containers. Therefore, we have not used the temperature of a single or several sterilized items as a criterion for determining readiness for unloading. The temperature of sterilized items decreases with the increment of time, and given the unique characteristic of time, we have selected time as the standard for unloading. Both Chinese and American sterilization national standards recommend a minimum cooling time of at least 30 minutes; hence, we have set the initial reminder time for our timing reminder device to 30 minutes [13,20]. Although the sterilized items loaded in each sterilization cycle may vary, including paper-plastic pouches, cloth-wrapped packages, rigid containers, and non-woven pouches, the mass and volume of each type of packaging vary only slightly. Additionally, the total load of items in each sterilization cycle has an upper limit. Therefore, selecting a cooling time suitable for the majority of sterilized items (30 minutes) is the most straightforward and reasonable approach. Even if the sterilized items are loaner instruments for orthopedic surgery (which are heavy and contain a significant amount of plastic material), SPD technicians may choose a specific sterilization cycle (with an extended vacuum drying phase at the final stage). This cycle ensures the dryness of the sterilized items as much as possible, making the 30-minute cooling threshold applicable in such cases as well.

**Fig 5:**
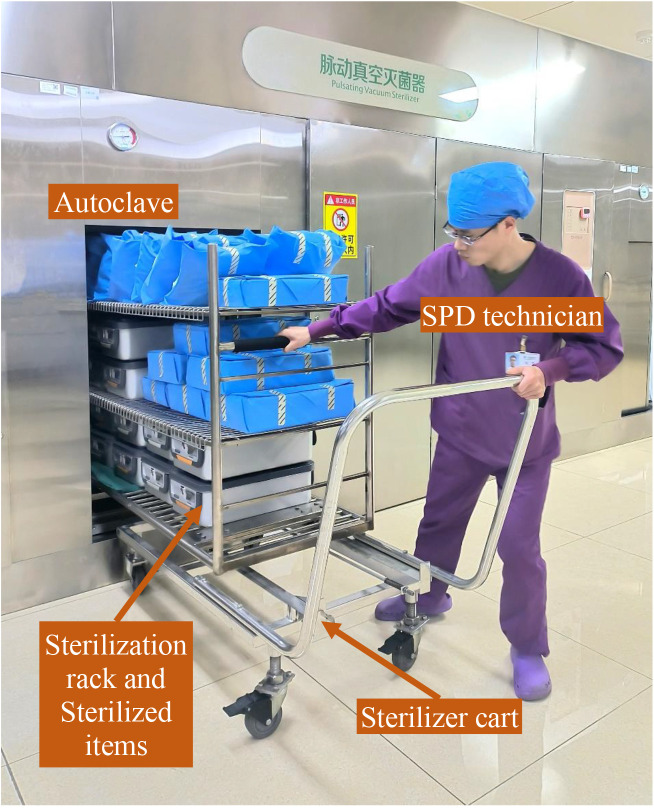
The SPD technician transfers the sterilization rack from the sterilizer chamber to a sterilizer cart.

**Fig 6:**
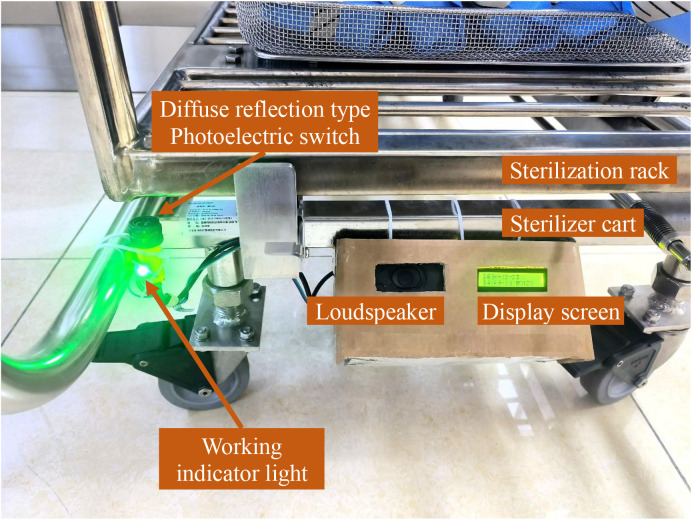
Installation of automatic timing reminder device on sterilizer cart. The photoelectric sensor switch (facing upward) and the working indicator light are secured to the sterilizer cart using plastic cable ties. The remaining components are consolidated into a single box, which is mounted beneath the cart. When the sterilization rack is positioned above the sensor, it triggers the sensor to start timing, and the working indicator light turns on and remains lit.

After the sterilization cycle is concluded, the SPD technician transfers the sterilization rack from the sterilizer chamber to a sterilizer cart, waiting the sterilized items to cool down on the cart (Fig 5). The photoelectric sensor switch and working indicator light of the device are secured to the sterilizer cart using plastic cable ties, with the sensor adjusted to face upward to ensure it can be activated by the sterilization rack. The remaining components are consolidated into a single box, which is mounted beneath the cart. The display screen is oriented toward the front and upper side, facilitating easy viewing by SPD technicians (Fig 6). This installation method offers significant flexibility, enhancing the device’s versatility and enabling its adaptation to a variety of equipment models.

After the fabrication and installation of this timing reminder device, it possesses the following functions:

a **Unobtrusive operation:** The photoelectric sensor switch of the timing reminder device is fixed on the sterilizer cart. The moment the sterilization rack is transferred onto the cart, it obstructs the sensor switch, initiating the timer from 00:00. Upon reaching the preset duration, the device begins playing music as a reminder. Once all sterilized items are unloaded onto the metal shelves, the SPD technician returns the empty sterilization rack to the steam sterilizer chamber, thereby releasing the obstruction on the sensor switch, and the device stops timing. The entire process operates automatically without requiring specific actions from the SPD technician.
b **Automatic reminders:** This device can provide multiple reminders during each operation, including an initial reminder and subsequent timed reminders. The first music reminder signals the start of unloading sterilized items, while subsequent music reminders indicate the need to expedite the unloading process. The start time of the initial reminder and music, the interval time for subsequent reminders and music, as well as the volume of the music, can all be customized.
c **Data management:** Data from each operation of this device is automatically stored. We can query the start time and duration of each timing device operation through a microSD card, facilitating SPD managers in managing and analyzing the cooling period of sterilized items and the work behavior of SPD technicians.

### Application of the device

Two identical autoclaves (Manufacturer: Shandong Xinhua Medical Instrument Co., Ltd.; Model: XG1.H; Chamber volume: 1.2 cubic meters) were used for testing, with one autoclave equipped with the timing reminder device serving as the experimental group, and the other unaltered autoclave serving as the control group. Considering that the use of the timing reminder device might significantly influence the behavior of SPD technicians, we set the testing period to 4 weeks. This timeframe avoided the Chinese Spring Festival (late January or early February) and the National Day (early October), ensuring that the 4-week period represented the typical workload conditions of the SPD. The testing was conducted within the SPD, where both temperature and humidity were controlled within appropriate ranges, showing no significant variations.

In the control group, after the completion of sterilization cycle, the SPD technician used a sterilizer cart to pull the sterilization rack out from the chamber and placed the cart in an area without air conditioning vents and with minimal personnel movement to allow the sterilized items to cool naturally. The SPD technician checked the current time on a wall clock, mentally noting the unloading time as “current time + 30 minutes.” When estimating that the unloading time had arrived, the SPD technician first performed hand hygiene and then determined whether to unload the sterilized items by touching them to sense their temperature. Once the items were unloaded, the sterilization rack was pushed back into the sterilizer chamber and the unloading door of the sterilizer was closed. The autoclave returned to standby mode. In the experimental group, the SPD technician’s operations were essentially the same as in the control group, but there was no need to estimate the unloading time, nor was it necessary to use the back of the hand to sense the temperature of the sterilized items. The SPD technician simply began unloading the sterilized items from the sterilizer cart to the metal shelves upon the musical reminder from the timing reminder device.

### Observation index

#### Cooling time compliance rate.

Cooling period refers to the interval from when sterilized items are pulled out of the chamber to the start of unloading. The number of times the cooling time of all sterilization cycles in two autoclaves reached 30 minutes was recorded, and the percentage of compliant cycles out of the total sterilization cycles was calculated. The stored video surveillance was reviewed to record the cooling time of the control group. Considering that the time stored by the timing reminder device in the experimental group included the time for unloading sterilized items and returning the sterilization rack to the chamber, video surveillance review was also used to record the cooling time of the experimental group. The following formula was used to complete the calculation:


$$Cooling\;Time\;Compliance\;Rate=\frac{Number\;of\;sterilization\;cycles\;with\;cooling\;time\;up\;to\;30\;minutes}{Total\;number\;of\;sterilization\;cycles}\times 100\;\%$$

#### Wet packs incidence rate.

Recorded the number of sterilized items from both autoclaves that exhibited wet packs during the testing period, and calculated the percentage of wet packs out of the total number. Information regarding wet pack was derived from feedback provided by SPD technicians, wards, and operating rooms. When SPD technicians were unloading, if they found beads of water or noticeable dampness on the exterior of instrument trays, paper – plastic packaging, or textile packages, these were recorded as wet packs. Similarly, when wards and operating rooms opened sterilized items and found beads of water inside the packaging, these were also recorded as wet packs. The following formula was employed for the purpose:

$Wet\;pack\;incidence\;rate=\frac{Number\;of\;wet\;packs}{Total\;number\;of\;sterilized\;items}$ *×100%*

#### Number of times SPD technicians touched sterilized items.

This metric observed how many times SPD technicians touched sterilized items with their hands during the cooling period before unloading. The fewer the touches, the better. We collected data by reviewing the surveillance records of the work area.

#### Satisfaction of SPD Technicians.

A self-designed satisfaction survey was administered to 5 SPD technicians (out of the 36 staff members in our SPD, only 5 hold the Chinese National Certification for Special Equipment Operators). The satisfaction survey included three items: ease of operation, safety and effectiveness, and potential for widespread adoption. Each item was scored using a Likert 5-point scale (1 = very dissatisfied, 2 = dissatisfied, 3 = moderately satisfied, 4 = satisfied, 5 = very satisfied).

### Statistical analysis

SPSS 23.0 was used for data analysis. Categorical data were statistically described using frequency and percentage, and intergroup comparisons were made using the chi-square test. Continuous data that did not conform to a normal distribution were represented as M (P_25_, P_75_), and intergroup comparisons were made using the Mann-Whitney *U* test. Statistically significant differences were identified if the p-value was less than 0.05.

## Results

### Comparison of cooling time compliance rate between two groups

The cooling time compliance rate of the experimental group was 92.93% (92/99), which was higher than the control group’s rate of 72.92% (70/96). The difference between the two groups was statistically significant, with χ2 = 13.884 and P < 0.001.

### Comparison of wet packs incidence rate between two groups

The wet packs incidence rate in the sterilized items of the experimental group was 0.18% (10/5651), which was lower than that of the control group at 0.49% (25/5138). The difference between the two groups was statistically significant, with χ^2^ = 7.978 and P = 0.005.

### Comparison of the number of times SPD technicians touch sterilized items between two groups

The experimental group and the control group conducted 99 and 96 sterilization cycles, respectively. The number of times the SPD technicians in the experimental group touched the sterilized items with hands before unloading was 1 (0, 1), which was fewer than the 3 (2, 4) times in the control group. The difference between the two groups was statistically significant, with *Z* = −10.172 and P < 0.001.

### Comparison of satisfaction between two groups of SPD technicians

The total satisfaction score in the experimental group, as well as the scores for the three items—ease of operation, safety and effectiveness, and potential for widespread adoption—were all higher than those in the control group (Table 1). The differences were statistically significant (P < 0.05).

**Table 1:** Comparison of satisfaction between two groups of SPD technicians (n = 5, M, (P_25_, P_75_)).

Groups	Total satisfaction score (Max. 15)	Ease of operation (Max. 5)	Safety and effectiveness (Max. 5)	Potential for widespread adoption (Max. 5)
Control group	6 (5, 7)	3 (2, 3)	2 (1.5, 2)	2 (1, 2.5)
Experimental group	14 (12.5, 14.5)	5 (4.5, 5)	4 (4, 4.5)	5 (4, 5)
Z value	−2.627	−2.739	−2.785	−2.668
P value	0.009	0.006	0.005	0.008

## Discussion

The occurrence of wet packs after autoclave is influenced by many factors, such as ambient temperature, the material of medical devices, the loading position of instrument trays and textiles, packaging materials, the quality of clean steam, the performance of the autoclave, sterilization cycle parameters, and cooling time [9,10,21]. Adequate cooling time is important to prevent the occurrence of wet packs. This is because the hot air inside the sterilized items, instrument trays, and rigid containers contains many water molecules. If they are immediately transferred to metal shelves, condensation due to the rapid cooling of hot materials can occur. At this time, sufficient cooling time is needed to allow the water molecules inside the packaging to transfer to the air outside the packaging in the form of water vapor, thereby reducing and avoiding the occurrence of wet packs [8]. The cooling time we have chosen is 30 minutes, which complies with the autoclave guidelines of both the United States and China. This choice is also aimed at improving the work efficiency of autoclave to start the next sterilization cycle as soon as possible [13,20]. Of course, the cooling time can be appropriately extended based on the actual situation on site.

### The device reduces the wet packs incidence rate by increasing the cooling time compliance rate

The control group relied on wall clocks and memory to determine the unloading time for sterilized items. This method is highly subjective and not conducive to standardizing the autoclave operation process. Additionally, sterilization staff may be busy with other tasks and forget to unload the sterilized items. The timing reminder device in the experimental group objectively records the cooling time and plays music to remind the SPD technician that they can start unloading sterilized items after 30 minutes. The experimental group’s compliance rate for cooling time was 92.93%, significantly higher than the control group’s 72.92%. In the experimental group, there were 7 sterilization cycles with cooling time less than 30 minutes, due to 5 cycles requiring very short cooling period (because the load was small, with fewer than 30 paper-plastic packages), and 2 cycles reprocessed instrument trays urgently needed in the operating room.

The increase in the cooling time compliance rate objectively provided more time for residual water molecules inside the instrument trays to transfer to the outside, thereby reducing the appearance of wet packs. If moisture or water droplets are found on the exterior packaging of sterilized items after the completion of the steam sterilization cycle in the SPD, these items need to be returned to the packaging area for re-drying and re-packaging before undergoing sterilization again. If wet packs are discovered inside the packaging on the operating table (water droplets inside the instrument trays can only be seen upon opening, which the SPD cannot detect), the surgical instrument trays must be replaced with new ones. Therefore, the occurrence of wet packs increases the cost of reprocessing instruments (including packaging costs, sterilization costs, labor costs, etc.) and disrupts the smooth progress of surgeries, causing inconvenience to patients, surgeons, and nurses [21]. The wet packs incidence rate in the experimental group was 0.18%, lower than that of the control group (0.49%) and also lower than the 0.26% reported by Dong [22] using an infrared gun to measure the cooling temperature of sterilized items.

### The device can reduce the potential contamination risks of sterilized items

The method used by the control group to determine the cooling time is highly subjective, and it also requires multiple instances of touching the surface temperature of the sterilized items with hands to judge the timing for unloading. Packages should not be touched until they are cool because a hand can act as a point of condensation for the warm water vapor emanating from the package, thereby creating a moist area on the outside of the package. This moist area can act as a wick to draw bacteria from the hands into the package [8]. Even though SPD technicians are required to perform hand hygiene each time before touching the sterilized items, compliance and correctness of hand hygiene cannot always reach 100%, and microorganisms on the hands may still enter the sterilized items through moist areas, causing contamination [23,24]. The timing reminder device in the experimental group can accurately time the cooling process, allowing SPD technicians to unload based on the device’s reminders, reducing the likelihood of repeatedly touching sterilized items during the cooling process, thereby reducing the potential contamination risks of sterilized items. Furthermore, the use of a timing reminder device can also reduce the workload of SPD technicians, including the frequency of hand hygiene, the number of times they touch sterilized items, and the number of steps taken while walking.

### The device has the potential for widespread application

The most notable innovation of the automatic timing reminder device is its automation. It enables unobtrusive operation, automatically starting and stopping the cooling time timer based on the presence or absence of the sterilization rack, without requiring any additional actions from the SPD technician. The device has a dual reminder function: it plays reminder music A when the timer reaches 30 minutes to indicate that the cooling time has been met and unloading can commence, and then plays reminder music B every 5 minutes thereafter to remind of the need to unload promptly. This ensures sufficient cooling time while also improving work efficiency. Thanks to its development based on embedded technology, the initial reminder time, the interval between reminders, different reminder music, and volumes can all be adjusted according to actual needs. The SPD technicians express high satisfaction with this device, finding it simple and convenient to operate, highly effective, and worthy of promotion and application across multiple SPDs.

The device is mounted on the sterilizer cart for use, secured with plastic cable ties. The plasticity and adaptability of plastic cable ties are extensive, meaning the timing reminder device has excellent compatibility and can be installed on sterilizer carts dedicated to any type of autoclave. This versatility makes it suitable for both large and small SPDs, regardless of their specific operational requirements. Additionally, different SPDs can set the initial reminder time based on the volume and mass of their sterilized items, as well as customize reminder music and intervals according to their departmental work habits. Most importantly, the device is cost-effective, priced at only 35 to 40 US dollars.

Beyond its automation and compatibility, another significant advantage of this device is its ability to record and store the cooling time for each sterilization cycle. Compared to the medical device cleaning and packaging areas, the pressure steam sterilization area is more independent, especially evident in larger SPDs. Independent work areas make it challenging to supervise the work behavior of SPD technicians. The use of the timing reminder device can automatically record the cooling time for each sterilization cycle, and this data can be stored long-term. To some extent, this helps standardize the work behavior of SPD technicians and contributes to the quality management of pressure steam sterilization processes.

To manage the cooling period of sterilized items effectively, some SPDs use manual timers or infrared guns to determine the appropriate unloading time. However, both methods have significant limitations. The manual timer method requires SPD technicians to manually start and stop it multiple times. Additionally, the reminder music of the manual timer cannot be changed, it cannot provide continuous reminders, and the recorded data cannot be stored. The infrared gun method is influenced by factors such as the volume of the items, packaging materials, and the distance between the gun and the sterilized items, which may result in significant discrepancies between the measured temperature and the actual temperature. Using the infrared gun also significantly increases the workload of SPD technicians, as they need to measure each sterilized item sequentially. Measuring items on the lowest layer of the sterilizer cart requires frequent bending, which increases the risk of fatigue and injury to the technicians’ lower back.

Overall, the automatic timing reminder device offers advantages such as automated operation, excellent compatibility, low cost, and data storage capabilities. It standardizes the management of cooling period for autoclave, reduces the wet packs incidence rate and the potential contamination risks of sterilized items, and improves the satisfaction of SPD technicians. Therefore, it is highly necessary to promote its widespread adoption in other SPDs.

### Limitations

The data from the automatic timing reminder device is stored on a microSD card, which requires a computer to export and view the records, making the process less convenient. In the future, we plan to integrate a wireless transmission module into the device, enabling the stored data to be transmitted to computers and mobile devices via a wireless network. Additionally, the device will provide warnings for sterilization cycles that fail to meet the cooling time requirements.

Since the device is fixed to a mobile sterilizer cart, it cannot be connected to a continuous power supply. Currently, two 5000mAh batteries are used alternately, with each battery providing approximately 60 hours of continuous operation. This requires sterilization technicians to replace the batteries periodically. Alternatively, the device’s battery life can be extended by using higher-capacity batteries.

## Conclusion

The automatic timing reminder device, developed based on embedded systems, demonstrates good cost-effectiveness and high satisfaction among SPD technicians, while also standardizing the management of autoclave. In the future, its limitations, such as battery life and inconvenient data transmission, will be gradually improved, and the device will be promoted for widespread adoption across multiple SPDs. This widespread adoption could significantly enhance the efficiency and safety of sterilization processes in healthcare settings. Embedded systems can also be applied in the future to areas such as automatic timing reminders for immersion disinfection, patient activity management, and supervision of staff behavior.

## Supporting information

S1 Research data. (XLSX)

S2 Code for the timing reminder device. (TXT)
